# Developing social capital in implementing a complex intervention: a process evaluation of the early implementation of a suicide prevention intervention in four European countries

**DOI:** 10.1186/1471-2458-13-158

**Published:** 2013-02-20

**Authors:** Fiona M Harris, Margaret Maxwell, Rory C O’Connor, James Coyne, Ella Arensman, András Székely, Ricardo Gusmão, Claire Coffey, Susana Costa, Zoltan Cserháti, Nicole Koburger, Chantal van Audenhove, David McDaid, Julia Maloney, Peeter Värnik, Ulrich Hegerl

**Affiliations:** 1 Nursing, Midwifery and Allied Health Professions Research Unit, Iris Murdoch Building, University of Stirling, FK9 4LA, Stirling, UK; 2 Suicidal Behaviour Research Laboratory, School of Natural Sciences, University of Stirling, FK9 4LA, Stirling, UK; 3 Department of Psychiatry, University of Pennsylvania School of Medicine, 3535 Market Street, 6th Floor, 19104, Philadelphia, PA, USA; 4 National Suicide Research Foundation, 1 Perrott Avenue, College Road, Cork, Ireland; 5 Institute of Behavioural Sciences, Semmelweis University Budapest, Nagyvárad tér 4, 1089, Budapest, Hungary; 6 CEDOC, Departamento de Saúde Mental, Faculdade de Ciências Médicas da Universidade Nova de Lisboa, 1169-056, Lisbon, Portugal; 7 Klinik und Poliklinik für Psychiatrie und Psychotherapie, Universitätsklinikum Leipzig AöR, Semmelweisstraße 10, 04103, Leipzig, Germany; 8 LUCAS, Katholieke Universiteit Leuven, Kapucijnenvoer 39 - bus 5310, 3000, Leuven, Belgium; 9 Personal Social Services Research Unit, London School of Economics and Political Science, Houghton Street, WC2A 2AE, London, UK; 10 Klinik und Poliklinik für Psychiatrie, Psychosomatik und Psychotherapie der Universität Würzburg, Füchsleinstraße 15, 97080, Würzburg, Germany; 11 Estonian-Swedish Mental Health and Suicidology Institute, Õie 39, 11615, Tallinn, Estonia

**Keywords:** Complex interventions, Process evaluation, Suicide prevention, Realist evaluation, Social capital, Advisory groups

## Abstract

**Background:**

Variation in the implementation of complex multilevel interventions can impact on their delivery and outcomes. Few suicide prevention interventions, especially multilevel interventions, have included evaluation of both the process of implementation as well as outcomes. Such evaluation is essential for the replication of interventions, for interpreting and understanding outcomes, and for improving implementation science. This paper reports on a process evaluation of the early implementation stage of an optimised suicide prevention programme (OSPI-Europe) implemented in four European countries.

**Methods:**

The process analysis was conducted within the framework of a realist evaluation methodology, and involved case studies of the process of implementation in four European countries. Datasets include: repeated questionnaires to track progress of implementation including delivery of individual activities and their intensity; serial interviews and focus groups with stakeholder groups; and detailed observations at OSPI implementation team meetings.

**Results:**

Analysis of local contexts in each of the four countries revealed that the advisory group was a key mechanism that had a substantial impact on the ease of implementation of OSPI interventions, particularly on their ability to recruit to training interventions. However, simply recruiting representatives of key organisations into an advisory group is not sufficient to achieve impact on the delivery of interventions. In order to maximise the potential of high level ‘gatekeepers’, it is necessary to first transform them into OSPI stakeholders. Motivations for OSPI participation as a stakeholder included: personal affinity with the shared goals and target groups within OSPI; the complementary and participatory nature of OSPI that adds value to pre-existing suicide prevention initiatives; and reciprocal reward for participants through access to the extended network capacity that organisations could accrue for themselves and their organisations from participation in OSPI.

**Conclusions:**

Exploring the role of advisory groups and the meaning of participation for these participants revealed some key areas for best practice in implementation: careful planning of the composition of the advisory group to access target groups; the importance of establishing common goals; the importance of acknowledging and complementing existing experience and activity; and facilitating an equivalence of benefit from network participation.

## Background

Developing interventions to prevent suicide and non-fatal suicidal acts is a major public health challenge in many countries [1,2]. Such interventions range from individual to multilevel interventions with the latter offering considerable promise [3]. However, evaluation of multilevel suicide prevention interventions, which are often driven by national suicide prevention programmes, is limited [4-7]. For example, although the Finnish and Scottish national suicide prevention programmes were evaluated, they adopted a broad approach to evaluation rather than focusing on the effects of the specific interventions [8,9]. The evaluation of the Finnish suicide prevention strategy concluded that the effort had not fostered the level of professional and political commitment required for sustainability; and projects were insufficiently integrated with mainstream health care systems. One of the aims of our multi-level suicide prevention intervention was thus to engage relevant regional stakeholders and create local, collaborative networks with the intention of planning for sustainable activity in the event that effectiveness of the intervention was demonstrated [10].

Based on lessons learned from the implementation of the European Alliance Against Depression (EAAD), we identified network capacity as having an important role to play in both the reach and implementation of the interventions. Key to the successful development of networks and network capacity, is the accrual of social capital [11,12] to the core of the network, which we conceptualise as our multi-level suicide prevention consortium. Social capital is defined as “the features of social organisation, such as networks, norms and trust that facilitate coordination and cooperation for mutual benefit” [12]: p35]. By extension, this social capital is then tapped into and shared by network participants.

The focus of an analysis informed by social capital is on the relationships between agents, institutions and so on and the notion of ‘value’ that is embedded in social relationships. For our purposes, this links well into an understanding of OSPI network capacity. In our study protocol [10] we hypothesised that the formation of advisory groups would facilitate implementation activity, therefore we focused on the role and function of advisory groups in each country to explore whether or not this was the case.

Drawing on process evaluation data, this paper explores the role of advisory groups in stakeholder engagement and how different models of engagement both influenced implementation and the potential for capacity building and sustainability of an optimised suicide prevention programme in four European countries (Optimised Suicide Prevention and Implementation in Europe: OSPI-Europe). We unpack the process of early implementation in more detail including: organisational structures, partnership/stakeholder roles and their potential impact on implementation.

### Optimising suicide prevention and its implementation in Europe (OSPI-Europe)

OSPI implemented five levels of suicide prevention interventions in Germany, Hungary, Ireland and Portugal, with a control and intervention site in each country. OSPI’s multilevel approach builds on the Nuremburg Alliance Against Depression [13,14] and the European Alliance Against Depression (EAAD) [3], which pioneered four of the levels of intervention: training for health professionals in primary care; public relations and mass media campaigns; training for those working in community settings who may come into contact with depressed and/or suicidal persons (such as teachers, members of the police force, social workers and so on); and support for self-help groups, high risk groups and their families. The fifth level, addressing access to lethal means was added to the OSPI approach, informed by evidence of best practice for suicide prevention. This level primarily involves identifying suicide hotspots and including information in training sessions for health care providers on the toxicity of certain drugs when taken in overdose.

A fuller discussion of the OSPI-Europe approach, including details on the primary and intermediate outcome measures are provided elsewhere [10]. This paper concentrates on the early implementation stages of approximately 18 month duration in each of the four intervention sites.

## Methods

The process evaluation was informed by realist evaluation methodology [15,16]. Realist evaluation places an emphasis on the importance of context within complex interventions, going beyond the evaluation question “What works?” to what works, for whom and in what context. Following Pawson and Tilley [15], it is clear that in order to understand what works in suicide prevention we have to pay attention to the complex social world where interventions are implemented.

When outcomes data for the OSPI interventions become available, these will be explored within the contexts in which they were achieved (drawing on macro-, meso-, and micro-level data). However, in this paper we explore processes of early implementation in order to understand what may have helped to achieve early implementation goals: including gaining access to a wide range of sectors for suicide awareness training, dissemination of public awareness campaign materials, the identification of at risk groups, and suicide hotspots. We therefore restrict reporting of methods to those relevant to this paper.

The aims of this paper are:

1. To identify the organisational and partnership structures which underpin early implementation activity.

2. Explore the mechanisms of engagement that promote active participation and collaboration in early phases of implementation.

### Data collection

The OSPI interventions took place in Germany, Hungary, Ireland and Portugal. Each country has an intervention and a comparison/control site. Each of the four research teams sought ethical review and gained approval from the relevant bodies in each country: Ethics Commission of the Medical Faculty, University of Leipzig, Germany (refs. 248-2007 and 140-2009-06072009); Semmelweis University Regional and Institutional Committee of Science and Research Ethics, Hungary (ref. TUKEB 149/2009), Ethics Research Committee of the Mid-West Regional Hospital, Limerick City and County, Ireland (no reference number, letter of approval dated 25/06/2009) and Clinical Research Ethics Committee, Merlin Park University Hospital, Galway City and County, Ireland (ref. C.A. 271); and the Ethical Committee of the Faculty of Medical Sciences, New University of Lisbon, Portugal (ref. CE/DP/7-2009).

We combined the following methods for our case study analysis: progress tracking questionnaires (exploring timing, delivery and intensity of implementation activities); interviews and focus groups with stakeholder groups; and observations at OSPI project team meetings.

Data on local contexts, including whom they involved in their local partnerships (advisory groups) and how they were taking forward local implementation plans were gathered via questionnaires, qualitative interviews and/or focus groups at six monthly intervals from January 2010. The fifth and final phase of data collection (consisting of workshops to explore local capacity and sustainability), was completed in September 2012. This paper reports on data from across three waves of data collection covering the early set up and implementation phases of OSPI. These data are supplemented by fieldnotes from participant observation at five OSPI Project meetings held during the implementation phase of the project.

Semi-structured interviews and focus groups were conducted with key stakeholders who have a role to play in local suicide prevention and/or implementation of the interventions in each of the four countries. They mostly included members of the local advisory groups or key individuals engaged in facilitating local implementation. Local researchers (trained by the process evaluation team) conducted the interviews and focus groups in the participants’ own languages. Interviews were recorded, transcribed verbatim and translated (where appropriate) into English. Quotes reported here are therefore close approximations of the verbatim recordings rather than exact replicas. Table 1 shows the completed numbers of interviews or focus groups conducted in each wave.

**Table 1:** Interviews and focus groups

	**Phase 1**	**Phase 2**	**Phase 3**	**Total**
**Germany**	6 interviews	1 focus group	8 interviews	14 interviews; 1 focus group
**Hungary**	10 interviews	1 focus group	1 focus group	10 interviews; 2 focus groups
**Ireland**	7 interviews	1 focus group	6 interviews	13 interviews; 1 focus group
**Portugal**	3 interviews	5 interviews	2 interviews	10 interviews
**Total interviews & focus groups**		47 interviews; 4 focus groups		

The qualitative data sought to situate the interventions within any local issues that might impinge on implementation of the 5 level activities, such as other national or local suicide prevention or depression awareness campaigns running alongside OSPI activities, or any major economic events such as large factory closures or other manifestations of the recession.

Participant observation was carried out at OSPI meetings by FH, with additional notes added by MM. Observations were recorded as fieldnotes [17] to supplement the minutes of the meetings. While minutes of meetings recorded progress within each of the intervention countries, our fieldnotes focused on issues related to the processes of implementation, paying particular attention to the barriers and facilitators to implementation experienced within each country. Furthermore, during these meetings FH was able to clarify any points that had arisen through interviews or focus groups with members of the various research teams.

### Data analysis

Each country was treated as a case study and data collection followed a longitudinal approach designed to capture the process of change [18]. Qualitative data were therefore analysed via a longitudinal, case study approach [19,20], drawing on techniques of framework analysis [21]. The interview, focus group and observational data were charted under thematic headings for each country, and a framework was developed to explore the barriers and facilitators to implementation. Both within-case and cross-case themes were identified via the framework method, which were then developed further using an interpretive approach. While we took a longitudinal approach to data collection and analysis, the material presented here mainly consists of thematic content that arose from the first set of interviews and continued to present and develop across the subsequent two waves of data collection. However, the impact of participation in advisory groups was explored longitudinally, allowing participants to reflect on the costs and benefits that OSPI involvement brought to their organisations.

In order to protect participant anonymity we present our results as Cases A-D.

## Results

Analysis of local contexts in each of the four countries revealed that the advisory group was a key mechanism that had a substantial impact on the ease of implementation of OSPI interventions. The advisory group was intended to facilitate implementation of OSPI activity but also brought stakeholders together, established or broadened partnership working among members and enhanced the potential for local capacity building in suicide prevention and the future sustainability of intervention activities. We tracked the development and participation of these groups over time. However, advisory group membership was fluid, contingent on implementation activities and organisations’ resources and could change over time.

First we will describe the four models of advisory group established in each intervention region and how they affected implementation, providing an example of this for two of the intervention levels. We also describe how successful engagement with OSPI activities requires the transformation of potential advisory group participants into OSPI stakeholders and how this transformation was facilitated, namely through:beliefs that the OSPI project and its leaders came with a positive history of prior achievement, and gave partners the sense they were involved in something bigger; personal affinity with the shared goals of OSPI (including the need for training); the participatory approach that sought to maximise and compliment local achievements; and the reciprocity of rewards for participant organisations through extending their networks and collaborations.

### The advisory groups and their impact on implementation

Advisory group members included a mix of representatives of professional groups (such as GPs or pharmacists) or organisations representing various health, social welfare and voluntary sector agencies at national/regional/local level.

Case A followed a specialist mental health/acute care model with a strong emphasis on psychiatry in advisory group representation. However, this was balanced by inclusion of representatives of the self-help movement, which has a strong presence in mental health care in this country. It is clear that while training was conducted across a range of sectors, particularly within medicine, this team also benefited from informal relationships with key gatekeepers across community sectors that facilitated recruitment into training within their professions.

Case B had a strong steer from a large, multi-disciplinary academic team, with the advisory group led by primary care with additional community involvement. From interviews and observations at OSPI meetings, it was clear that this advisory group had no difficulty engaging with primary care. For instance, as a GP who was interviewed revealed, both his father and brother were GPs with some involvement in the implementation of training in primary care in this intervention region.

*First my father was asked to be involved in OSPI, and then he asked me to join. Since this year I took over the further training of GPs from him, it means that I have good contact with colleagues here, which is very important. […] This is how I can help the programme* (Case B, Interview 1-1).

It was clear that the Case B team had identified and engaged a family of GPs who were influential in primary care in the region, which facilitated uptake of OSPI training by GPs.

Case C had an interdisciplinary advisory group, retaining a strong participatory, community based approach to the development and implementation of the interventions. The group act as high level ‘gatekeepers’ into a wide range of sectors that have a role to play in suicide prevention, including health, education, social work, the police force, members of the clergy and so on. The emphasis on community organisations facilitated access to a wide range of community settings with uptake of training from a range of sectors, particularly the police. On the other hand, they had a slower uptake from GPs in this intervention region, partly due to existing similar training initiatives.

Case D had no formal advisory group and this team spoke of cultural difficulties in bringing different professional sectors together. The system called for formal protocols to be agreed upon prior to accessing each organisation (including the community sector). They developed a small number of informal relationships with gatekeepers. Rather than a collaborative model of working, this team was constrained by a hierarchical bureaucracy and formal protocols that was less conducive to research practices. Furthermore, the OSPI team found it difficult to engage health professionals, many of whom had a perceived lack of capacity to commit to OSPI: ‘*so when we tried to speak with the people in charge, the first response was like “Oh no, more work, more things that we have to do, more demands on my time”, you know?’* (Case D, Interview 1-3).

**Figure 1:**
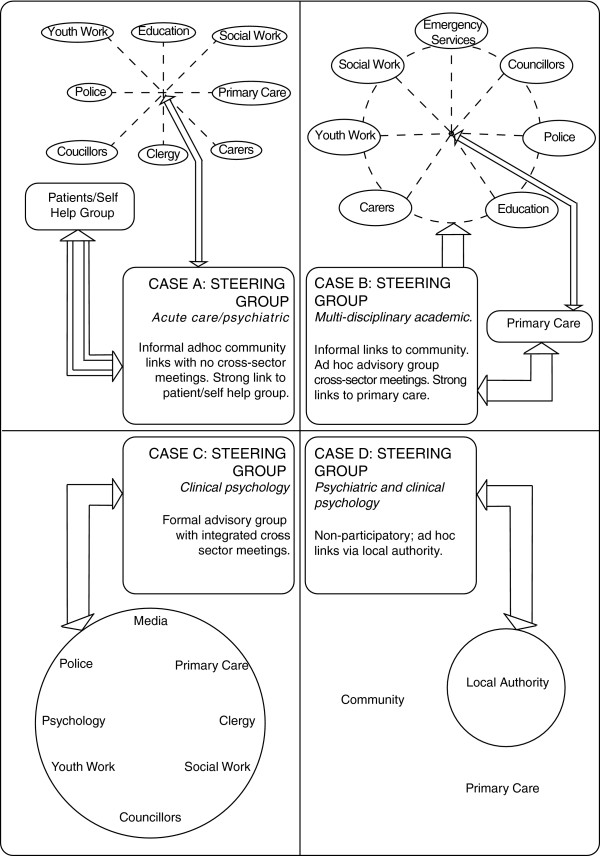
Models of steering and advisory groups.

The advisory group (whether a concrete group or a virtual one that relied on informal, ad hoc contact), acted as a mechanism that played a crucial role in implementation: facilitating access to different professional groups for training, and members often acting as both advisors and gatekeepers across a range of sectors as Figure 1 illustrates. Thus, the reach of the group was important to implementation processes.

One area where the influence of the advisory groups was especially important was in recruitment to both GP (level 1) and community facilitator (level 3) training. Although all four countries trained the target numbers of GP’s (proportionate to the population sizes of the intervention regions), data collection at six monthly intervals revealed variation in the length of time it took to recruit and train General Practitioner’s in Primary Care (GP’s) as Table 2 illustrates. Cases A and B experienced less difficulty compared to Cases C and D, because the former had strong links with local GP champions, whereas although Case C had GP representation on their advisory group, they did not have a local level of influence. Added to this, similar GP training had already taken place in this site, therefore OSPI training was seen as a duplication of effort. This was resolved by adapting the OSPI training into a short refresher course, which was delivered some months later than originally planned. Case D relied on local authority connections that did not have strong links with primary care. After much delay, they achieved the target numbers of GP’s by resorting to political manoeuvring. Their head of psychiatric services reached an agreement with their counterpart in primary care and issued a protocol that made participation in OSPI training a mandatory activity for all available GPs. This contrasts with the voluntary and collaborative approach of other regions and is likely to have limited sustainable delivery of training in the future as we describe in more detail below.

**Table 2:** GP/other medical settings training

**LEVEL 1: GP/other medical training**	**09-12/2009**	**01-03/2010**	**04-06/2010**	**07-09/2010**	**10-12/2010**	**01-03/2011**	**04-06/2011**	**07-09/2011**
**Case A**	32 GP	32 GP	45 GP	16 Acute ward staff	19 Ambulance staff; 14 Midwives			
**Case B**		50 GP	10 Clin Psychols		74 Nurses	5 GP	30 Nurses	
						48 Nurses		
**Case C**					11 GP		7 GP	80 GP
Case D*			18 GP	11 Nurses	46 GP	7 GP		
			23 Nurses					
			5 Clin Psychols					
			3 Primary Care Social Workers					

*An additional 10 workshops were conducted with general hospital staff as additional activity that was not part of the evaluation but nevertheless added value to OSPI suicide prevention activity.

Table 2 illustrates the timing and roll-out of Level 1 GP training. Target numbers for training (as well as target size and intensity of the public awareness campaign materials) were calculated prior to inception of the implementation phase, based on previous studies, so local teams were given targets proportionate to the population size in each intervention site.

Table 3 shows the variation in community-based professionals that were trained (a Level 3 activity). In exploring this variation, our analysis identified the local advisory groups as the key mechanism to facilitate recruitment and uptake of training interventions. Cases A, B, and C involved a wide range of community sectors in training compared to Case D, who were unable to develop strong, community-based links and experienced long delays in implementing training and had a limited number of community sectors involved. Partnership and cross-sector working were not part of the culture and Case D was further hindered by the need for formal, signed protocols and agreements to enable access to potential training recruits.

*And [name] is saying that it’s difficult to bring different professionals like psychiatrists together with social workers because they don’t work together very well … it’s the hierarchy, it’s difficult* (Case D, Interview 3-2).

*Because we want to run the training and we can’t. Without this* [agreement] *we can’t because we have to respect the bureaucracy* (Case D, Interview 1-3).

**Table 3:** Community facilitator training

LEVEL 3: community facilitator training*	**09-12/2009**	**01-03/2010**	**04-06/2010**	**07-09/2010**	**10-12/2010**	**01-03/2011**	**04-06/2011**	**07-09/2011**
**Case A**	12 Pol; 16 CLC; 12 Ph;	122 Pol; 25 SW; 74 T; 79 Ca; 26 Ph; 26 MS	83 SW; 58 T; 4 Ca; 13 Ph; 12 Cler; 13 HI;	14 YW; 65 SW; 28 T; 27 Ca; 13 Cler; 11 MS	10 SW; 51 Ca; 11 Cler; 16 HI; 10 ST			
**Case B**			20 YW; 70 SW; 30 T; 14 Pol; 5 CLC; 5 Ca; 11 Cl	50 Ph; 53 Cler	42 T;	30 YW; 82 T;	9 YW; 50 T; 35 Ca; 9 Others (country specific); 8 CBT Traing	
**Case C**			100 Pol	10 YW; 200 Pol	100 Pol	12 SW; 8 Probation Officers	37 Cler	10 CLC; 16 Ph
**Case D**						11 Cler	7 SW; 10 Jour	12 Cler
							16 CPsy	302 Pol (Oct-Dec 2011)
							3 Ca	
							1 YW	
							1 Soc	
							1 SS	
							6 T	

*“Community facilitators” are community-based professionals. Abbreviations are as follows:

*YW*, Youth workers; *SW*, Social/community workers; *T*, Teachers; *Pol*, Police; *Jour*, Journalists; *CLC*, Crisis Line Counsellors; *Ca*, Carers for the elderly; *Ph*, Pharmacists; *Cler*, Clergy (all faiths); *HI*, Health Insurance Staff; *MS*, Medical Secretaries; *ST*, Sports Trainers; *EAS*, Employment Agency Staff; *Soc*, Sociologist; *CPsy*, Community Psychol; *SS*, Social secretaries.

Furthermore, the advisory group members assisted the public information campaign (level 2 activity) by acting as channels for dissemination, helping to identify local suicide hotspots (one of the concerns of level 5), and helped in the development of initiatives for those at risk of suicide (level 4). Thus, they were key to implementation activity in all of the five intervention levels.

### Transformative engagement: from passive to active participation in OSPI

Simply recruiting representatives of key organisations into an advisory group was not sufficient to achieve their buy-in to helping with the delivery of interventions. These representatives were often senior members within organisations with their own organisational objectives and OSPI could have been seen as an additional burden they did not need to undertake. They could also have participated purely in an ‘advisory’ capacity without any commitment to undertake OSPI tasks such as participation in training. It became clear from our experience of implementation in different countries that in order to engage and maximise the potential of high level ‘gatekeepers’ (who simply facilitated access to organisations), it was necessary to first transform them into OSPI stakeholders.

We explored the issue of an ‘engaged’ advisory group by asking interviewees (who were advisory group members) about their reasons for wanting to be involved in OSPI. Some of our interviewees spoke of the prior history of the OSPI consortium and how this had encouraged them to participate in an advisory capacity. Many participants had heard of the prior work of the European Alliance Against Depression and felt that OSPI was a positive further development of this.

*I know that they achieved a 40% decrease in suicide in* [city name] *The* […] *result made me very enthusiastic, especially because I know that they were able to maintain this level the following year as well* (Case B, Interview 6-1).

Furthermore, this positive history was capitalised on by some of the OSPI teams so that in Case A, for instance, they promoted all of their OSPI activities under the banner of the Alliance Against Depression rather than OSPI. In other words, they continued to build on and draw on the reputation of the Alliance Against Depression.

The perception that OSPI was an evidence-based suicide prevention programme driven by academics was highlighted as important by some interviewees. Similarly, one interview participant was interested in the possibility that there might be lessons to learn that he could draw on from wider European contexts: ‘*I feel that it’s important that we have European linkages’* (Case C, Interview 1-1)*.*

Indeed, it is likely that the prestige of having both a European-wide consortium as well as EU Framework 7 funding potentially made involvement in OSPI even more attractive. Furthermore, in the current economic climate of scarce resources and cuts in healthcare funding, participants could point to OSPI as an exemplar project, to argue that suicide prevention activities were worthy of continued support. Added to the pan-European collaboration and the opportunity for learning from this, was also the possibility that advisory group members might gain a sense of being part of something that transcended the local and linked them into a much larger suicide prevention community.

While prestige and wider learning opportunities may have motivated some, an additional and widely acknowledged further incentive for becoming involved in OSPI was the participatory approach to implementing the interventions adopted in three of the four countries. Rather than simply developing new initiatives and imposing them on pre-existing suicide prevention strategies or other interventions, in fact the OSPI interventions were designed to maximise and complement what was already in place. Indeed, several interviewees commented that OSPI ‘added value’ to local initiatives, and reflected shared goals between their organisations goals and those of OSPI. As one advisory group member commented: “*I saw a very strong linkage between this project and the* [local] *strategic development in mental health* [in Case C] (Case C, Interview 1-1).

Another incentive to take part in OSPI was the locally recognised need to train professionals across various sectors to help them identify individuals at risk of suicide as well as to help them to deal with suicidal acts. For instance, one focus group participant highlighted the trauma to professionals attending the scene of a suicide attempt or indeed a death by suicide without having adequate training.

*Every member out of the 498 trained… I’d say 400 of them would have some involvement* [in suicide] *and there would be stories from talking to a young man on a cliff for three hours and eventually he said sorry and jumped. Some really bad cases like that … to police going into places seeing people hanging from rafters and trees and so on. So, in our organisation that training was badly needed and that’s why we’re involved in this today* (Case C, Focus Group 1-2).

Finally, the advisory group extends the local research teams’ networks into organisations where interventions are to take place. For instance, in Case C, members of the advisory group have facilitated access to a range of professional groups for training sessions, extending the reach of OSPI community facilitator training sessions (Level 3 activity) across a wide range of sectors that may regularly come into contact with individuals at higher risk of suicidal behaviour, such as drugs action, ethnic minority health, the youth work service and so on. Advisory group members were also responsible for distributing awareness raising materials (Level 2, Public awareness campaign) through their own networks.

Advisory group meetings themselves may bring people from different sectors together for the first time, which may facilitate the development of cross sector networks for each of the members. Advisory group members who were interviewed spoke about how they were able to exchange information and expertise, sometimes with unexpected consequences that enhanced the common goal of suicide prevention. For instance, in Case C, advisory group members from the police force met with a representative from a local organisation responsible for river safety, which then led to developing a new collaboration and joint initiative aimed at reducing deaths by drowning (Level 5, reducing access to lethal means). The advisory group thus acts as a kind of ‘network bridge’ that allows members’ access to expertise across a range of sectors that they may not come into close contact with on a routine basis.

*.... I suppose for the others on the panel they get to meet people from.... let’s say the addiction services and all of the other services that are out there* […]. *So obviously the networking for everyone involved is good* […]. *Sometimes people don’t even know what resources are out there so through the advisory panel people would have learned of a lot that was going on* (Case C, Interview 5-3)*.*

However, only three of the four countries could be described as achieving the establishment of advisory groups that had fully engaged and collaborative partners. Whilst the required intensity of most OSPI activities was eventually achieved by all (through sustained efforts and some delays) there were marked differences in the achievement of ‘optional’ activities which required substantially more input from external partner organisations. For instance, the suicide awareness and prevention training provided by OSPI includes a ‘train the trainer’ component. This involves providing training to key professionals that they can then roll out more widely within their respective organisations [22,23]. The ‘train the trainer’ model helps to plan for a sustainable increase in local capacity in suicide prevention, with at least the potential for training interventions to continue beyond the life of the funded project. Tellingly, Case D, did not achieve the transformative relationships needed with their implementation partners and were the only country that did not implement any ‘train the trainer’ sessions.

However, simply increasing capacity via training trainers is not enough to produce a sustainable training programme. As one of the interviewees in Case B noted:

*I think that’s what we saw in EAAD, was that after we left* […] *then everything went back to the same level as it was before* (Case B, Interview 10-1).

This interviewee revealed that without any management structure or plan being put in place to steer the continued roll-out of training sessions, nothing was taken forward and the momentum generated by the new capacity was lost. Other advisory group members also recognised this and suggested that the advisory group itself might be harnessed to continue to manage the roll-out and support of OSPI training interventions after the end of the project which demonstrates the level of transformation not only to stakeholder status but to potential ‘ownership’ status.

*when* [OSPI Lead] *is gone, we’re all still going to be here and we should be looking at maybe how we can … sustain and maintain* (Case C, Interview 4-1)*.*

While the advisory group and a participatory approach to implementation extended the reach of OSPI and produced positive interactions between different sectors with an interest in suicide prevention, there is also a cautionary tale from one intervention region. This team (like other OSPI groups) engaged the local media and received media attention both locally and nationally (a Level 2, Public campaign activity). Added to this was an intensive public awareness campaign of posters, leaflets and so on, which carried news of OSPI activities extensively across the implementation region. The rollout of training across primary care and community sectors added to this OSPI-related activity. However, it later transpired that the high visibility of OSPI came at a price.

*I suppose what’s very disappointing for us is that we have delivered a huge amount of awareness training before OSPI came to* [intervention city] *and we have delivered a huge amount of skills-based training* […] *and it’s like people have forgotten they ever did it because now the best thing that ever came is OSPI* (Case C, Interview 5-3).

This interviewee spoke of how the considerable work done by their agency had received negative comments that questioned their (local) expertise and capacity in suicide prevention. This person reported feeling that the local community saw the OSPI academic team as coming to the rescue, rather than supporting and extending what was already in place. This perception was reported despite the fact that the OSPI team had emphasised at various events and training sessions that they were adding to local capacity rather than bringing something new.

## Discussion

Theories of social capital enhanced our understanding of both the four intervention site contexts as well as the mechanisms that promoted participation and engagement by advisory group partners. For instance, by exploring the range of advisory group participants and understanding their motivation to engage with OSPI activities, we were able to gain an insight into what facilitated recruitment into suicide awareness and prevention training. It was clear that the inclusion of a wide range of sectors within an advisory group was a way of gaining access to a range of sectors and to develop reciprocity of benefit. The OSPI teams gained implementation capacity through ease of access to target sectors, advisory group members provided local expertise, and organisational capacity to ensure that appropriate staff were trained, providing premises and other in-kind assistance that makes implementation activities easier to achieve. Advisory group members benefited from achieving personal or organisational goals in suicide prevention; and extended their own networks and partnerships. A collaborative model where all partners benefited was adopted successfully in three of the four countries. In the fourth site (Case D), local cultural patterns of working prevented taking this model forward. While they achieved their target numbers of trainings and public campaign dissemination, this was achieved with greater effort. Furthermore, without accessing professionals to undertake the ‘train the trainer’ sessions, capacity will remain the same at the end of the intervention. Thus, an engaged local group of stakeholders, brought together in advisory groups appeared to be a key component that offers the potential for reciprocity, capacity building and sustainability.

Drawing on theories of social capital enables us to extend our understanding of the processes that facilitate ‘engagement’ in OSPI interventions. Putnam [12,24] differentiated between two different kinds of social capital: bonding and bridging social capital. In the ‘bonding’ form of social capital, like-minded groups are drawn together to form strong supportive links, whereas ‘bridging’ social capital is regarded as the capital accrued by bringing together heterogeneous groups. While the former is stronger and more enduring, Putnam argues that bridging social capital is nevertheless more likely to promote inclusion. Thus, the focus of an analysis informed by social capital is on the relationships between actors, institutions and so on and the notion of ‘value’ that is embedded in social relationships.

While Putnam [12,24] presents bonding and bridging social capital as a dichotomy, in operationalising these terms with regard to OSPI networks, we find that rather than two distinct typologies, bonding and bridging social capital might be more usefully regarded as a continuum. While the core OSPI network (that is the OSPI research team within each country) might be regarded as having bonding social capital, nevertheless the extended network created via the advisory group might better be conceptualised as having bridging social capital – particularly within groups such as Cases B and C where there are a range of health and community sectors represented. However, clearly over time these groups shared more common ground and a common purpose and one might argue that what began as bridging capital (enhanced by the common goal of suicide prevention) eventually transformed into bonding social capital, thus developing a firmly engaged advisory group/implementation team.

The advisory groups accrue value and social capital by association with the OSPI consortium. The social capital that is attributable to OSPI has its origins in pre-existing networks first formed during the implementation of the interventions associated with the European Alliance Against Depression. In each of the four countries, a local Alliance Against Depression had implemented the four level suicide prevention approach that was one of the pre-cursors to OSPI, albeit not in the same region where the OSPI intervention took place. The perceived success of this Alliance meant that OSPI activities were enhanced by the social capital already embedded in this country’s team. Furthermore, as suggested above, being part of a pan-European consortium funded by EU Framework 7 also generated social value for OSPI researchers. Participants in advisory groups could thus tap into this social capital, transcending the local implementation by feeling part of a European network. In turn, this sharing of social capital enhanced engagement, thus ensuring that the advisory group increased the reach of OSPI interventions via their own extensive networks.

In at least one implementation country (Case C), OSPI accrued a large amount of social capital through media attention, the public campaign and the extensive rollout of training. However, despite a participatory approach which led to strong buy-in from existing suicide prevention agencies and a wide range of community partners, the positive ‘publicity’ accrued from the collective action was seen to undermine or dilute previous local initiatives in suicide prevention. This experience was not reported from any other case study sites but the potential to lose or dilute organisational credit for activity to another collective may lead to questioning future or longer term participation. It seemed that in this example at least, social capital was a finite resource and while OSPI absorbed a large amount of social capital, this was to the detriment of local agencies, whose own perceived social value declined in the process. Even though the negative comment came from only one source, the interviewee who reported this clearly used this example to communicate feelings that local services had somehow lost value as OSPI activities gained in visibility.

### Implications for implementing complex interventions

Our results to date have wider implications for the implementation of complex interventions. Firstly, intervention teams should consider planning advisory group membership involving key sectors of relevance prior to the launch of interventions. In order to maximise the reach of the intervention and ease of implementation, representation from a wide range of health and community sectors should be considered. Providing opportunities for organisations to meet with each other via advisory group meeting will be more likely to ensure reciprocal benefits.

We suggest that attention should be paid to fully engaging members in order to ensure that they have a ‘stake’ in the intervention and thus, the intervention team can tap into their expertise and wider networks for the benefit of the delivery of the intervention. In OSPI, we achieved this by working with local initiatives and complementing pre-existing activities/programmes rather than imposing entirely new developments. This complementary/participatory approach ensured that OSPI activities were perceived as ‘adding value’, with an equivalence of benefit. However, it is also important to emphasise the value of local services at every opportunity, both publicly and otherwise.

It may be worth considering at the outset how the advisory group might become a management team that could continue to deliver the intervention beyond the life of the project, thus ensuring a degree of sustainability if the intervention demonstrates effectiveness. While the OSPI programme aims to build local capacity in suicide prevention and awareness via a ‘train the trainer’ model, nevertheless, we have also learned from previous experience of EAAD that in order for an intervention to truly be sustainable, there is a need to also implement or encourage a management structure that can continue to guide and plan for a continuation of activities beyond the life of the project.

## Conclusion

Theories of social capital afford a more nuanced picture of the processes inherent in early implementation of complex interventions. Taking a longitudinal approach to our analysis has allowed us to go beyond the more usual retrospective or ahistorical approach to evaluation to situate OSPI activities within networks and social capital that links to a pan-European suicide prevention agenda. Exploring the composition of advisory groups and the meaning of participation for these particular actors has revealed the importance of strengthening network capacity for successful reach and implementation of the interventions. Although the analysis presented here of early implementation processes is important in its own right, we will extend this when outcome data from the OSPI evaluation become available.

### Recommendations for implementation practice

Carefully plan the composition of advisory group membership to maximise implementation and sustainability of a suicide prevention programme.

Make use of personal experience and affinity with suicide prevention to express common goals.

Efforts to maintain reciprocity of benefit must include shared recognition of achievements by all individual partners. This may be facilitated with the transformation from ‘gatekeeper’ status to ‘stakeholder/ownership’ status of the suicide prevention programme.

The chance of longer term sustainability of interventions will be improved if local partners are encouraged to develop ‘ownership status’ for any intervention.

Acknowledge existing experience, expertise and activity already achieved by suicide prevention stakeholders and aim for complementarity. This may require flexibility in interventions or target groups.

Recognise the need for reciprocity of benefits in participation.

Recognise and promote opportunities for networking amongst group members to achieve added value from participation for the programme, for group members themselves and the organisations that they represent.

## Competing interests

In the last three years UH received honoraria as speaker or advisor from Lilly, Wyeth, Lundbeck, Bristol-Myers Squibb, Takeda and Sanofi-Aventis. He was also a consultant for Nycomed. All other authors declare that they have no competing interests.

## Authors’ contributions

FH co-ordinated data collection, analysis and the writing of this paper; MM designed and led the study and contributed to the analysis; ROC and JC contributed to the study design and analysis; EA, RG and AS contributed to the intervention design and led the intervention in Ireland, Portugal and Hungary; CC, SC, ZC and NK collected data; UH was the principal investigator for OSPI-Europe and led the intervention in Germany. All authors were involved in writing this paper and approving the final manuscript.

## Pre-publication history

The pre-publication history for this paper can be accessed here:

http://www.biomedcentral.com/1471-2458/13/158/prepub
